# Adrenergic receptors blockade alleviates dexamethasone-induced neurotoxicity in adult male Wistar rats: Distinct effects on β-arrestin2 expression and molecular markers of neural injury

**DOI:** 10.1007/s40199-023-00490-y

**Published:** 2023-11-15

**Authors:** Rasha M. S. M. Mohamed, Enssaf Ahmad Ahmad, Dalia M. Amin, Samar Ahmed Abdo, Islam A. A. E.-H. Ibrahim, Mona F. Mahmoud, Shimaa Abdelaal

**Affiliations:** 1 Department of Clinical Pharmacology, Faculty of Medicine, Zagazig University, Zagazig, 44519 Egypt; 2 Department of Human Anatomy and Embryology, Faculty of Medicine, Zagazig University, Zagazig, 44519 Egypt; 3 Department of Forensic Medicine and Clinical Toxicology, Faculty of Medicine, Zagazig University, Zagazig, 44519 Egypt; 4 Department of Biochemistry, Faculty of Veterinary Medicine, Zagazig University, Zagazig, 44519 Egypt; 5 Department of Pharmacology and Toxicology, Faculty of Pharmacy, Zagazig University, Zagazig, 44519 Egypt

**Keywords:** Dexamethasone, Neurotoxicity, Carvedilol, Propranolol, Doxazosin, β-Arrestin2

## Abstract

**Background:**

Dexamethasone-induced neurotoxicity has been previously reported. However, the molecular mechanisms are still not completely understood.

**Objectives:**

The current work aimed to investigate the modulatory effects of α- and β-adrenergic receptors on dexamethasone-induced neurotoxicity in rats focused on changes in β-arrestin2 and molecular markers of neural injury in cerebral cortex.

**Methods:**

Male Wistar rats were subcutaneously injected with dexamethasone (10 mg/kg/day) for 7 days to induce neural injury in the cerebral cortex. The experiment involved 5 groups: control, dexamethasone, carvedilol, propranolol, and doxazosin. In the last 3 groups, drugs were given 2 hours before dexamethasone injection. At the end of experiment, brain samples were collected for measurement of brain derived neurotrophic factor (BDNF), glial fibrillary acidic protein (GFAP), kinase activity of protein kinase B (Akt), diacylglycerol (DAG), α-smooth muscle actin (α-SMA), Smad3, β-amyloid and phospho-tau protein levels in addition to histopathological examination of brain tissue using hematoxylin-eosin, Nissl, and Sirius red stains. Moreover, β-arrestin2 levels in the cerebral cortex were measured using immunohistochemical examination.

**Results:**

Dexamethasone slightly reduced brain weight and significantly decreased BDNF, Akt kinase activity and β-arrestin2 but markedly induced degeneration of cortical neurons and significantly increased GFAP, DAG, α-SMA, Smad3, β-amyloid and phospho-tau protein levels compared to controls. Carvedilol, propranolol, and doxazosin reversed all dexamethasone-induced molecular changes and slightly ameliorated the histopathological changes. Carvedilol significantly increased brain weight and β-arrestin2 levels compared to dexamethasone, propranolol, and doxazosin groups.

**Conclusion:**

blocking α- and/or β-adrenergic receptors alleviate dexamethasone-induced neurotoxicity despite their distinct effects on β-arrestin2 levels in the cerebral cortex.

**Graphical abstract:**

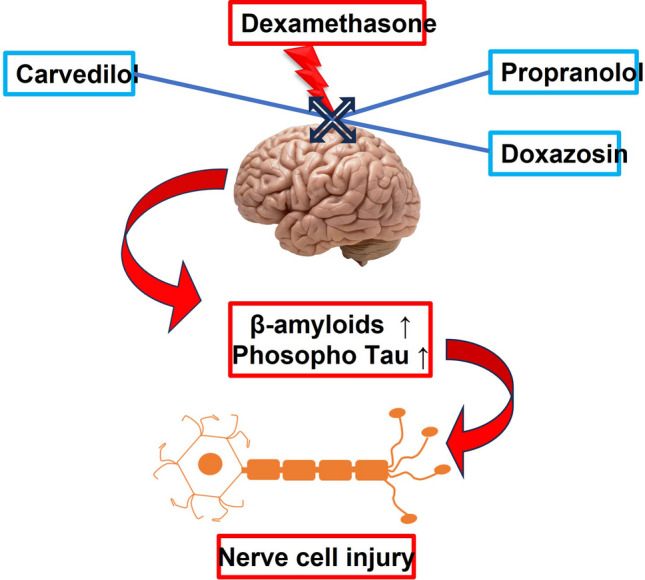

## Introduction

Dexamethasone is a fluorinated steroid that has distinct glucocorticoid activity. Dexamethasone has anti-inflammatory activity 25-times higher than hydrocortisone and longer duration of action [1]. Notably, dexamethasone is widely used as an anti-inflammatory and immunosuppressive medication [2]. In addition, dexamethasone is used in the management of chemotherapy-induced nausea and vomiting [3], and metastatic spinal cord compression [4]. The latter use needs high dexamethasone dose up to 100 mg which may cause serious side effects [5].

Noteworthy, dexamethasone but not hydrocortisone has been found to induce neurotoxicity in the neonatal rat brain. The neurotoxicity involved reduction of brain weight gain, increased caspase-3 activity, and number of apoptotic cells in addition to impaired learning and memory capabilities [6]. Similarly, administration of dexamethasone early or later after birth in preterm infants to treat bronchopulmonary dysplasia has been shown to increase brain lesions and neuromotor deficits [7].

Adrenergic receptors including β- and α-subtypes are G-protein-coupled receptors (GPCRs) that are expressed in most body tissues including the cerebral cortex [8]. α-Adrenergic receptors (α-ARs), especially α1-subtype, play a key role in cognition and memory. High dose of α1-AR agonist has been found to impair cognition in contrast to therapeutic doses which improve cognition and memory [9]. In the same context, β1-ARs play a significant role in both synaptic plasticity and memory formation. Furthermore, β2-ARs have been found to play a role in hippocampus-dependent long-term memory formation and processing [10].

β-Arrestin2, also named arrestin-3, is a GPCR desensitizing and scaffolding protein. Accumulating evidence suggests a potential neuroprotective role of β-arrestin2 in the brain. β-Arrestin2 mediates anti-inflammatory effects and reduces activation of microglia thereby ameliorating neuronal loss [11]. The neuroprotective effect of amisulpride, atypical antipsychotic and dopamine 2 receptor antagonist, has been found to be β-arrestin2 dependent [12]. Moreover, sevoflurane-induced neuronal apoptosis has been reduced by activation of β-arrestin2 signaling [13].

Carvedilol and propranolol are non-selective β-blockers with distinct β-arrestin biased agonistic effects [14, 15]. They are clinically used as anti-hypertensive medications, prophylaxis against esophageal variceal bleeding, and in the treatment of chronic heart failure [16]. Notably, previous studies showed neuroprotective effects for both drugs [17–19]. Carvedilol has been found to promote neuroprotection against cerebral ischemia, stroke, and diabetic neuropathy by mediating both anti-hypertensive and antioxidant effects [17, 18]. On the other hand, propranolol has been found to exert neuroprotective effects on retinal injury models [19].

Doxazosin is a selective α1-AR blocker which is clinically used as an anti-hypertensive medication and to treat lower urinary tract symptoms of benign prostatic hypertrophy [16]. Recently, doxazosin has shown neuroprotective effects on an in vitro model of Alzheimer's disease [20].

The current study aimed to identify the molecular mechanisms of dexamethasone-induced neurotoxicity in rats and the possible modulatory effects of adrenergic receptors blockade focusing on the changes in β-arrestin2 expression and levels of molecular markers of neural injury in the cerebral cortex.

## Materials and methods

### Animals

Adult male Wistar albino rats (180 ± 20 g, 8 weeks old) were purchased and housed in plastic cages with wood shavings as bedding in the animal care unit of our institution. The animals were kept under controlled temperature (23 ± 2 °C), humidity (60% ± 10%), and a 12-h/12-h light/dark cycle. Rats were acclimatized for at least two weeks prior to experiments with ad libitum access to standard pellet chow and tap water.

### Drugs and chemicals

Dexamethasone, propranolol, and doxazosin were obtained from EPICO Co. (10^th^ of Ramadan, Egypt). Carvedilol was obtained from Multi-Apex Pharmaceutical Co. (Cairo, Egypt). Dimethyl sulfoxide (DMSO) was obtained from Sigma-Aldrich (St. Louis, MO, USA). All drugs and chemicals used are of analytical grade (≥98%).

### Experimental design

After the acclimatization period, Rats were randomly divided into five experimental groups (n = 9 each). In group 1, Rats were given the vehicle (DMSO: Tween 80: Water in a volume ratio of 1:1:8) for 7 days [21]. In groups 2 to 5, rats were given dexamethasone (10 mg/kg/day, S.C., [22]). This dose is comparable to the dexamethasone dose used in humans (100 mg) for management of spinal cord compression [5]. Group 3: Rats received carvedilol (10 mg/kg/day, I.P.,[14]). The dose of carvedilol used in the current study is also comparable to that used in humans (100 mg) but for management of mild to moderate heart failure [23]. Group 4: Rats received propranolol (30 mg/kg/day, I.P., [24]). The Propranolol dose used in the present study is pharmacologically equivalent to that of carvedilol [25]. Group 5: Rats received doxazosin (5 mg/kg/day, I.P.,[26]). This doxazosin dose is equivalent to 50 mg dose in humans which is 3 times higher than the maximum dose used in humans (16 mg) for management of hypertension [27]. All drugs were dissolved in DMSO: Tween 80: Water in a volume ratio of 1:1:8 and given for 7 days, 2 hours before dexamethasone injection. In all groups, the injection volume was 500 μL per 200 g body weight.

### Tissue sampling

At the end of drug treatment, rats were euthanized by decapitation. Brain samples were collected. Brain samples were divided into two parts; one part was fixed and stored in 10% formalin for histopathological and immunohistochemical examination while the other was snap frozen in liquid nitrogen and stored at −80 °C for subsequent biochemical analyses.

#### Measurement of brain-weight and tibial length

At the end of drug treatment and before euthanasia, rats were fasted overnight, and tibial length was measured. After euthanasia, the brain was dissected and weighed.

#### Histopathological examination

Brain tissue was excised, fixed in 10% formalin, dehydrated in gradient ethanol, cleared in xylene, embedded in paraffin blocks, sectioned at 5-μm thickness, and stained with Hematoxylin and Eosin (H&E) stain, Nissel stain or Sirius red stain**.**

#### Measurement of cortical thickness

From each group, ten random cerebral cortical thickness linear measures were taken from all around the cerebral hemisphere, from 2 consecutive cut sections (each is 4µm thickness).

#### Calculation of fibrotic area using Sirius red stain

The relative collagen fiber staining area (% red area) was measured in brain sections using ImageJ v.1.51d (NIH & LOCI, Wisconsin University, USA). Briefly, fiber-positive areas were selected, masked by red binary coloring, and measured relative to a standard measurement frame.

#### Immunohistochemical detection of cerebral cortex β-arrestin2

An immunohistochemical study was performed utilizing the avidin-biotin-peroxidase technique [28]. Briefly, sections of 5μm were deparaffinized, rehydrated, washed in tap water, and suspended in 3% H2O2 for 10 min to inhibit the endogenous peroxidase. Trypsin (2%) was added to the tissue sections at 37°C for 10 min to induce the affinity for immune peroxidase staining technique. The tissue sections were placed in a solution of (10 mmol/l sodium citrate cushion, pH 6) then placed inside the microwave for 20 min for heat induction of antigen recovery. A blocking solution of phosphate-buffered saline (PBS) and 10% normal goat serum is added to block nonspecific protein binding. Using PBS, the diluted primary antibody was added to the slides, and they were incubated for 30 min, then few drops of streptavidin peroxidase were added to the slides, waiting for 20 min, then washed with PBS for 5 min. The chromogen Diaminobenzidine (DAB) (Dakopatts, Glostrup, Denmark) was added to the slides; then washed with distilled water. Finally, the slides were counterstained with Harri's hematoxylin (H), dehydrated, and cover slipped. Negative controls were run consistently in parallel by skipping the primary antibody.

β-Arrestin2 immunohistochemical staining appeared as brownish discoloration of the cellular cytoplasm. The primary antibody was the monoclonal antibody to β-arrestin2 (β-arrestin 2 (C16D9) Rabbit mAb, Cat No. #3857, Cell Signaling, Danvers, USA).

β-Arrestin2 immuno-stained sections were morphometrically analyzed. The area percentage of immune reaction to β-arrestin2 was measured within brain sections of 3 rats in each group at a magnification X 100 using ImageJ v.1.51d (NIH & LOCI, Wisconsin University, USA). Briefly, stain-positive (brown-colored) areas were selected, masked by red binary coloring, and measured relative to a standard measurement frame.

#### Measurement of biochemical changes in the cerebral cortex

Levels of brain derived neurotrophic factor (BDNF), glial fibrillary acidic protein (GFAP), kinase activity of protein kinase B (Akt), diacylglycerol (DAG), α-smooth muscle actin (α-SMA), Smad3, β-amyloids and phospho-tau protein were measured using ELISA kits supplied by MyBioSource (San Diego, USA, Cat. No. MBS355345), CUSABIO (Houston, TX, USA, Cat. No. CSB E08602r), CD Creative Diagnostics (Shirley NY, USA, Cat. No. DEIABL547), MyBioSource (San Diego, USA, Cat. No. MBS750727), MyBioSource (San Diego, USA, Cat. No. MBS266620), LifeSpan BioSciences (Seattle WA, USA, Cat. No. LS-F21581), MyBioSource (San Diego, USA, Cat. No. MBS702915), and Elabscience (Wuhan, China, Cat. No. E-EL-R1090) respectively. All procedures were performed according to the manufacturer instructions.

### Pooling of samples

Samples of each group were randomly pooled in three pools [29]. Each pool consists of either one, two or three samples based on the number of animals that survived to the end of the experiment.

### Statistical analysis

All data are presented as the mean ± standard error of the mean (SEM). Group means were compared by one-way ANOVA followed by Bonferroni post-test for selected pairs using GraphPad Prism v. 5 (GraphPad Software, Inc., La Jolla, CA, USA). A *p* < 0.05 was considered significant for all tests.

## Results

### Carvedilol increased brain weight and survival rate in dexamethasone-treated rats

**Fig. 1:**
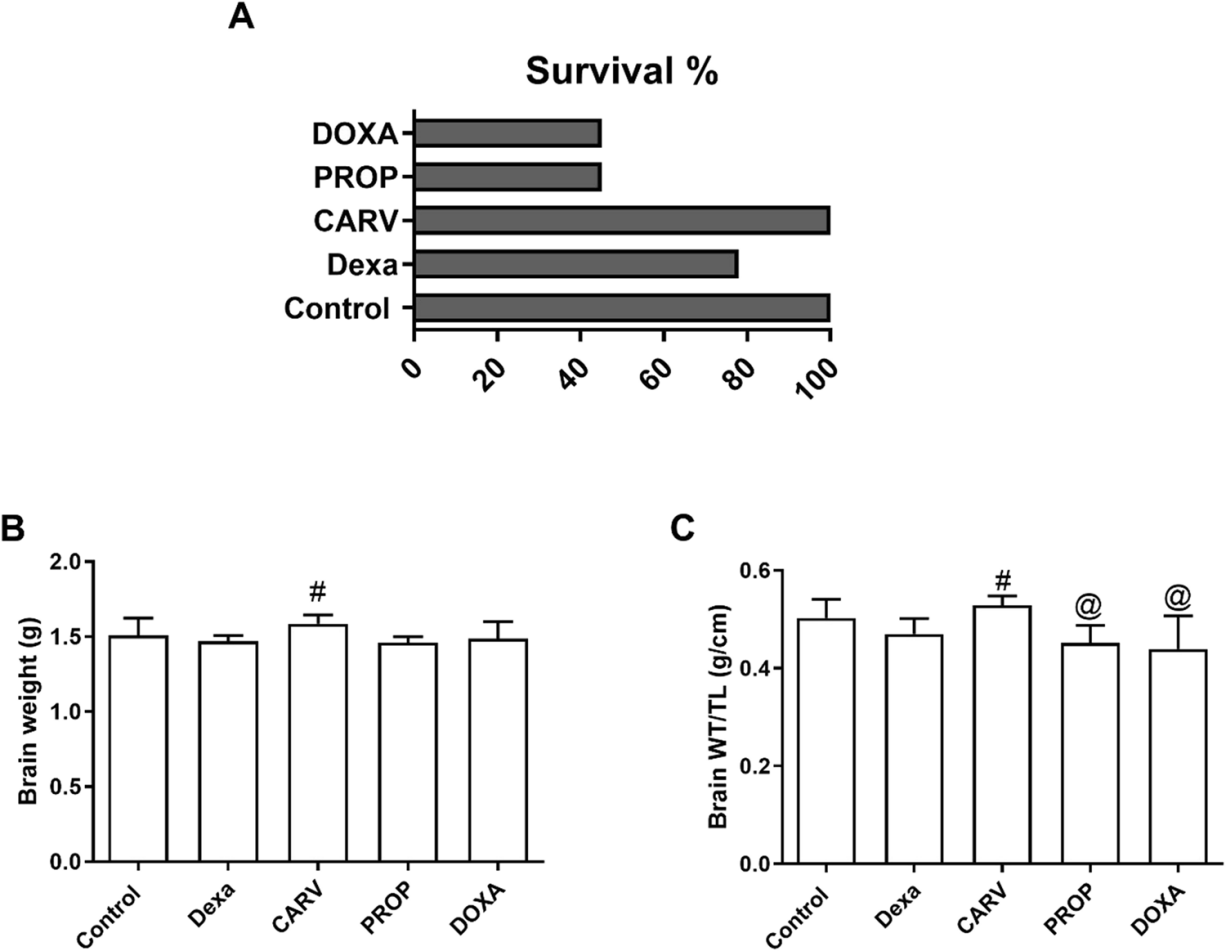
Changes in survival rate and brain weight. Graphical presentation of survival rate (**A**), brain weight (**B**), and brain weight normalized to tibial length (**C**). Control: Rats received vehicle for 7 days. DEXA: Rats received dexamethasone (10 mg/kg, S.C.) for 7 days. CARV: Rats received carvedilol (10 mg/kg, I.P.) then dexamethasone 2 hours later for 7 days. PROP: Rats received propranolol (30 mg/kg, I.P.) then dexamethasone 2 hours later for 7 days. DOXA: Rats received doxazosin (5 mg/kg, I.P.) then dexamethasone 2 hours later for 7 days. Statistical analysis was performed using one way ANOVA followed by Bonferroni post-test for selected pairs. Values are expressed as mean ± S.E.M. n=9 for the control and CARV groups. n=7 for the DEXA group. n=4 for the PROP, and DOXA groups. Samples of each group were randomly pooled in 3 different pools. # *p* < 0.05 vs Dexa; @ *p* < 0.05 vs CARV

As shown in Fig. 1, subcutaneous injection of dexamethasone (10 mg/kg/day) for 7 days reduced survival rate (78% vs 100%, Fig. 1A), brain weight (1.47±0.02 vs 1.5±0.04 g, F_4,27_=2.78, *p* = 0.046, Fig. 1B), and brain weight normalized to tibial length (0.47±0.01 vs 0.5±0.01 g/cm, F_4,27_=6.05, *p* = 0.0013, Fig. 1C) compared to control group. Carvedilol treatment increased survival rate (28%, Fig. 1A), significantly increased brain weight (8%, Fig. 1B) and brain weight normalized to tibial length (13%, Fig. 1C) compared to dexamethasone group and significantly increased survival rate (125%, Fig. 1A) and brain weight normalized to tibial length (18% and 20%, respectively, Fig. 1C) compared to propranolol and doxazosin treated groups.

### Carvedilol, propranolol, and doxazosin increased brain derived neurotrophic factor (BDNF) and Akt kinase activity and decreased glial fibrillary acidic protein (GFAP) and diacylglycerol (DAG) in the cerebral cortex of dexamethasone-treated rats

**Fig. 2:**
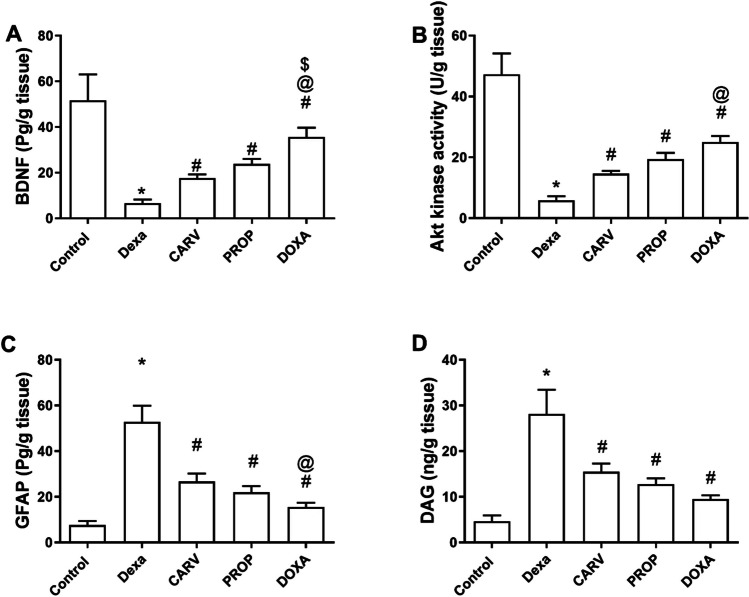
Changes in the cerebral cortex levels of brain derived neurotrophic factor (BDNF), Akt kinase activity, glial fibrillary acidic protein (GFAP), and diacylglycerol (DAG). Quantitative analysis and graphical presentation of BDNF (**A**), Akt kinase activity (**B**), GFAP (**C**), and DAG (**D**). Control: Rats received vehicle for 7 days. DEXA: Rats received dexamethasone (10 mg/kg, S.C.) for 7 days. CARV: Rats received carvedilol (10 mg/kg, I.P.) then dexamethasone 2 hours later for 7 days. PROP: Rats received propranolol (30 mg/kg, I.P.) then dexamethasone 2 hours later for 7 days. DOXA: Rats received doxazosin (5 mg/kg, I.P.) then dexamethasone 2 hours later for 7 days. Statistical analysis was performed using one way ANOVA followed by Bonferroni post-test for selected pairs. Values are expressed as mean ± S.E.M. n=9 for the control and CARV groups. n=7 for the DEXA group. n=4 for the PROP, and DOXA groups. Samples of each group were randomly pooled in 3 different pools. * *p* < 0.05 vs Control; # *p* < 0.05 vs Dexa; @ *p* < 0.05 vs CARV; $ *p* < 0.05 vs PROP

As estimated from Fig. 2, dexamethasone significantly decreased BDNF (6.6±0.9 vs 51.6±6.6 pg/g tissue, F_4,10_=28.65, *p* < 0.0001, Fig. 2A), increased GFAP (52.9±4 vs 7.6±1 pg/g tissue, F_4,10_=58.22, *p* < 0.0001, Fig. 2C), decreased Akt kinase activity (5.8±0.8 vs 47.2±4 U/g tissue, F_4,10_=62.83, *p* < 0.0001, Fig. 2B), and increased DAG (28.1±3 vs 4.6±0.7 ng/g tissue, F_4,10_=32.85, *p* < 0.0001, Fig. 2D) compared to control group. Notably, carvedilol, propranolol, and doxazosin treatments significantly increased BDNF (166%, 260%, and 437% respectively, Fig. 2A), decreased GFAP (49.5%, 58%, and 70% respectively, Fig. 2C), increased Akt kinase activity (150%, 230%, and 328% respectively, Fig. 2B), and decreased DAG (45%, 55%, and 66% respectively, Fig. 2D) compared to dexamethasone-treated rats. Furthermore, doxazosin significantly increased BDNF (102%, Fig. 2A), decreased GFAP (42%, Fig. 2C), and increased Akt kinase activity (71%, Fig. 2B) compared to carvedilol-treated rats.

### Carvedilol, propranolol, and doxazosin decreased α-smooth muscle actin (α-SMA), SMAD3, β-amyloid (1-42), and phospho-Tau protein in the cerebral cortex of dexamethasone-treated rats

**Fig. 3:**
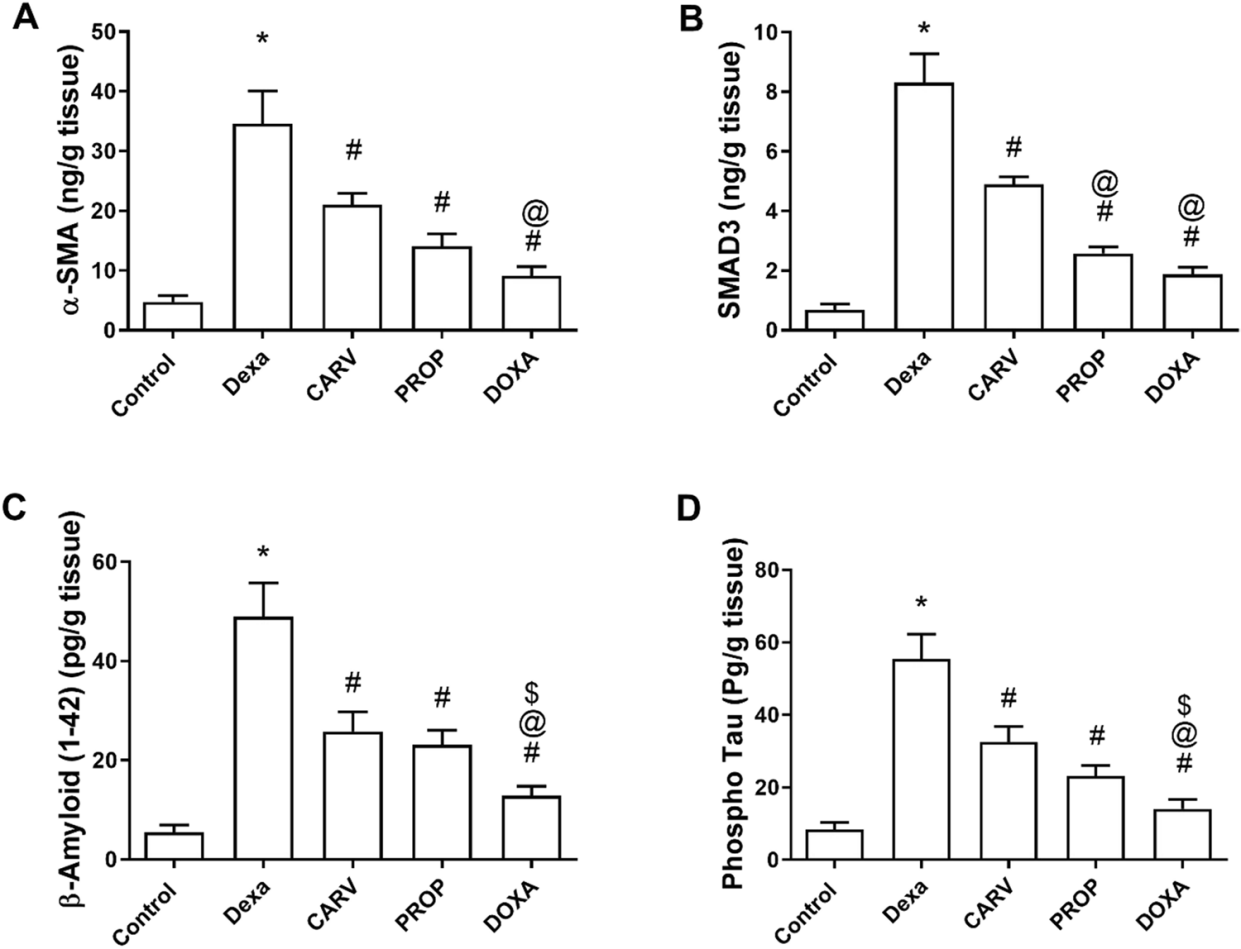
Changes in the cerebral cortex levels of alpha smooth muscle actin (α-SMA), SMAD3, β-amyloids (1-42), and phospho-Tau protein. Quantitative analysis and graphical presentation of α-SMA (**A**), SMAD3 (**B**), β-amyloids (1-42) (**C**), and phospho-Tau protein (**D**). Control: Rats received vehicle for 7 days. DEXA: Rats received dexamethasone (10 mg/kg, S.C.) for 7 days. CARV: Rats received carvedilol (10 mg/kg, I.P.) then dexamethasone 2 hours later for 7 days. PROP: Rats received propranolol (30 mg/kg, I.P.) then dexamethasone 2 hours later for 7 days. DOXA: Rats received doxazosin (5 mg/kg, I.P.) then dexamethasone 2 hours later for 7 days. Statistical analysis was performed using one way ANOVA followed by Bonferroni post-test for selected pairs. Values are expressed as mean ± S.E.M. n=9 for the control and CARV groups. n=7 for the DEXA group. n=4 for the PROP, and DOXA groups. Samples of each group were randomly pooled in 3 different pools. * *p* < 0.05 vs Control; # *p* < 0.05 vs Dexa; @ *p* < 0.05 vs CARV; $ *p* < 0.05 vs PROP

In Fig. 3, dexamethasone treatment significantly increased α-SMA (34.6±3 vs 4.7±0.6 ng/g tissue, F_4,10_=49.77, *p* < 0.0001, Fig. 3A), SMAD3 (8.3±0.5 vs 0.68±0.1 ng/g tissue, F_4,10_=119.7, *p* < 0.0001, Fig. 3B), β-amyloid (1-42) (48.9±3.9 vs 5.4±0.9 pg/g tissue, F_4,10_=53.72, *p* < 0.0001, Fig. 3C), and phospho-Tau protein (55.4±3.9 vs 8.4±1.1 pg/g tissue, F_4,10_=60.03, *p* < 0.0001, Fig. 3D) compared to control group. While carvedilol, propranolol, and doxazosin treatments significantly decreased α-SMA (39%, 59%, and 74% respectively, Fig. 3A), SMAD3 (41%, 69%, and 77% respectively, Fig. 3B), β-amyloid (1-42) (47%, 53%, and 74% respectively, Fig. 3C), and phospho-Tau protein (41%, 58%, and 74% respectively, Fig. 3D) compared to dexamethasone-treated rats. Furthermore, doxazosin significantly decreased α-SMA (56%, Fig. 3A), SMAD3 (61%, Fig. 3B), β-amyloid (1-42) (50%, Fig. 3C), and phospho-Tau protein (57%, Fig. 3D) compared to carvedilol-treated rats.

### Carvedilol increased cortical thickness of dexamethasone treated rats compared to propranolol and doxazosin groups

**Fig. 4:**
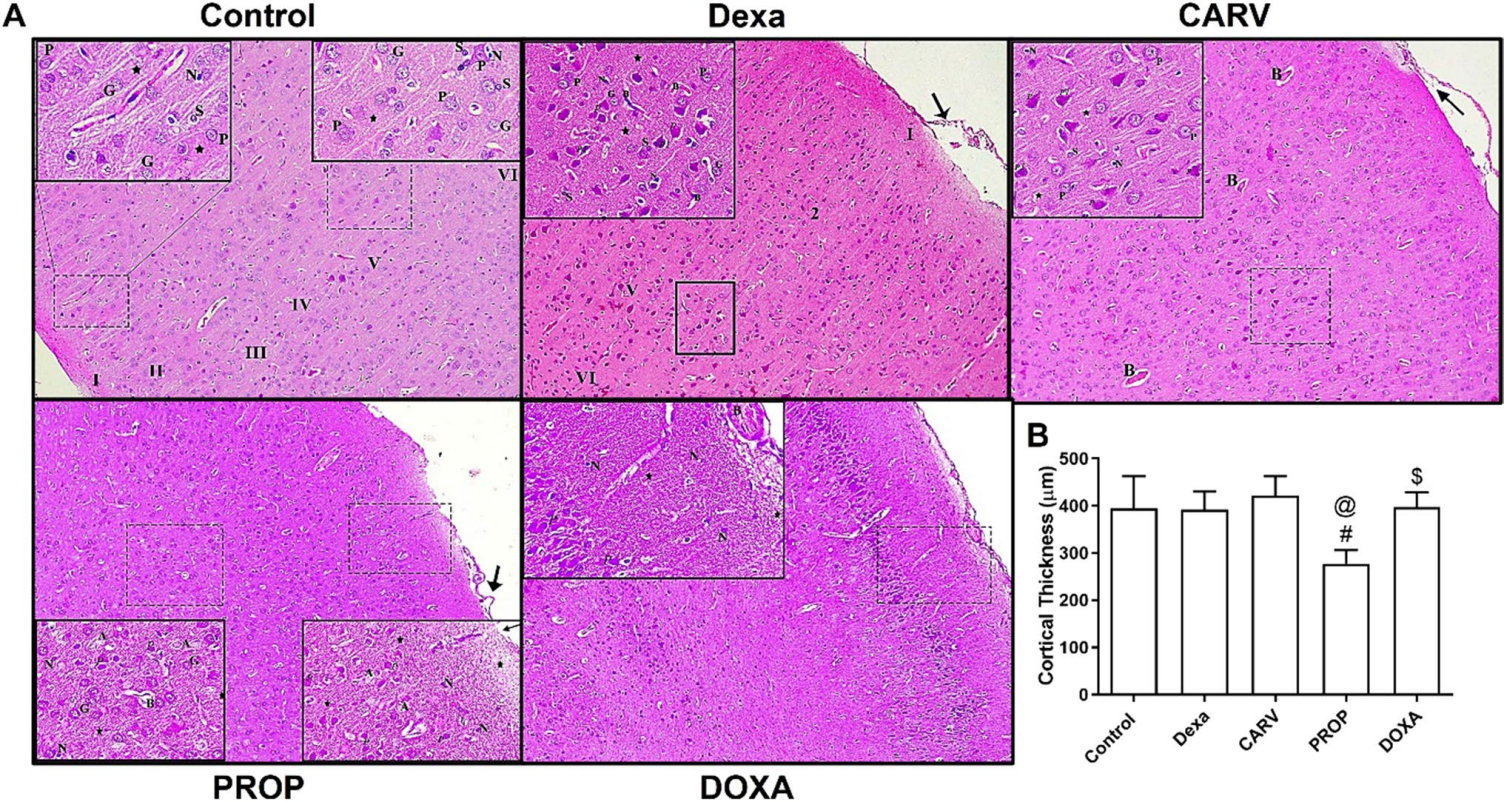
Histopathological examination of cerebral cortex using hematoxylin and eosin (H&E) stain. Photomicrographs of cerebral cortex sections stained with H&E (100x) (**A**). Graphical presentation of cortical thickness (**B**). Control: Rats received vehicle for 7 days. DEXA: Rats received dexamethasone (10 mg/kg, S.C.) for 7 days. CARV: Rats received carvedilol (10 mg/kg, I.P.) then dexamethasone 2 hours later for 7 days. PROP: Rats received propranolol (30 mg/kg, I.P.) then dexamethasone 2 hours later for 7 days. DOXA: Rats received doxazosin (5 mg/kg, I.P.) then dexamethasone 2 hours later for 7 days. I: molecular layer; II: outer granular layer; III: outer pyramidal layer; IV: inner granular layer; V: inner pyramidal layer; VI: pleomorphic layer; Star: neuropil; G: granular cells; S: astrocytes; N: neuroglial cells; Arrow: detached pia matter; B: blood vessels; **B**: Congested dilated blood vessels *P*: multiple scattered, dark stained, irregular pyramidal cells (degenerating neurons); P: normal pyramidal cells; *P1*: pyramidal cells with dark nuclei and wide pericellular halos (degenerating neurons). Statistical analysis was performed using one way ANOVA followed by Bonferroni post-test for selected pairs. Values are expressed as mean ± S.E.M. n=20 for all groups. From each group, ten random cerebral cortical thickness linear measures were taken from all around the cerebral hemisphere, from 2 consecutive cut sections # *p* < 0.05 vs Dexa; @ *p* < 0.05 vs CARV; $ *p* < 0.05 vs PROP

**Fig. 5:**
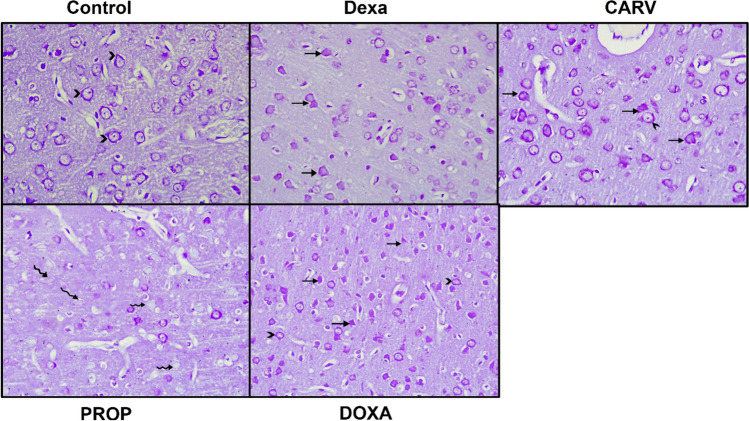
Histopathological examination of cerebral cortex using Nissl stain. Photomicrographs of Nissl-stained sections of cerebral cortex (400x). Control: Rats received vehicle for 7 days. DEXA: Rats received dexamethasone (10 mg/kg, S.C.) for 7 days. CARV: Rats received carvedilol (10 mg/kg, I.P.) then dexamethasone 2 hours later for 7 days. PROP: Rats received propranolol (30 mg/kg, I.P.) then dexamethasone 2 hours later for 7 days. DOXA: Rats received doxazosin (5 mg/kg, I.P.) then dexamethasone 2 hours later for 7 days. Arrowhead: Nissl granules surrounding vesicular nucleus. Arrows: Shrunken darkly stained pyramidal cells lacking their nuclei (degenerating neurons). Zigzag arrow: atrophic scanty stained shrunken cells lacking nucleus (degenerating neurons)

As shown in Fig. 4 and 5. subcutaneous injection of dexamethasone for 7 days caused marked disruption in the cortical layer integrity and degeneration of neurons (Fig. 4 and 5) without significant change in the cortical layer thickness compared to the control group (F_4,95_=33.38, *p* < 0.0001, Fig. 4.). Furthermore, carvedilol, propranolol, and doxazosin treatments slightly reduced the histopathological changes in dexamethasone-treated rats as estimated from Nissl-stained sections (Fig. 5). However, propranolol and doxazosin treatments significantly reduced the cortical thickness compared to the carvedilol group (21% and 10% respectively, Fig. 4B).

#### Neither dexamethasone nor treatments caused changes in fibrosis area in the cerebral cortex

**Fig. 6:**
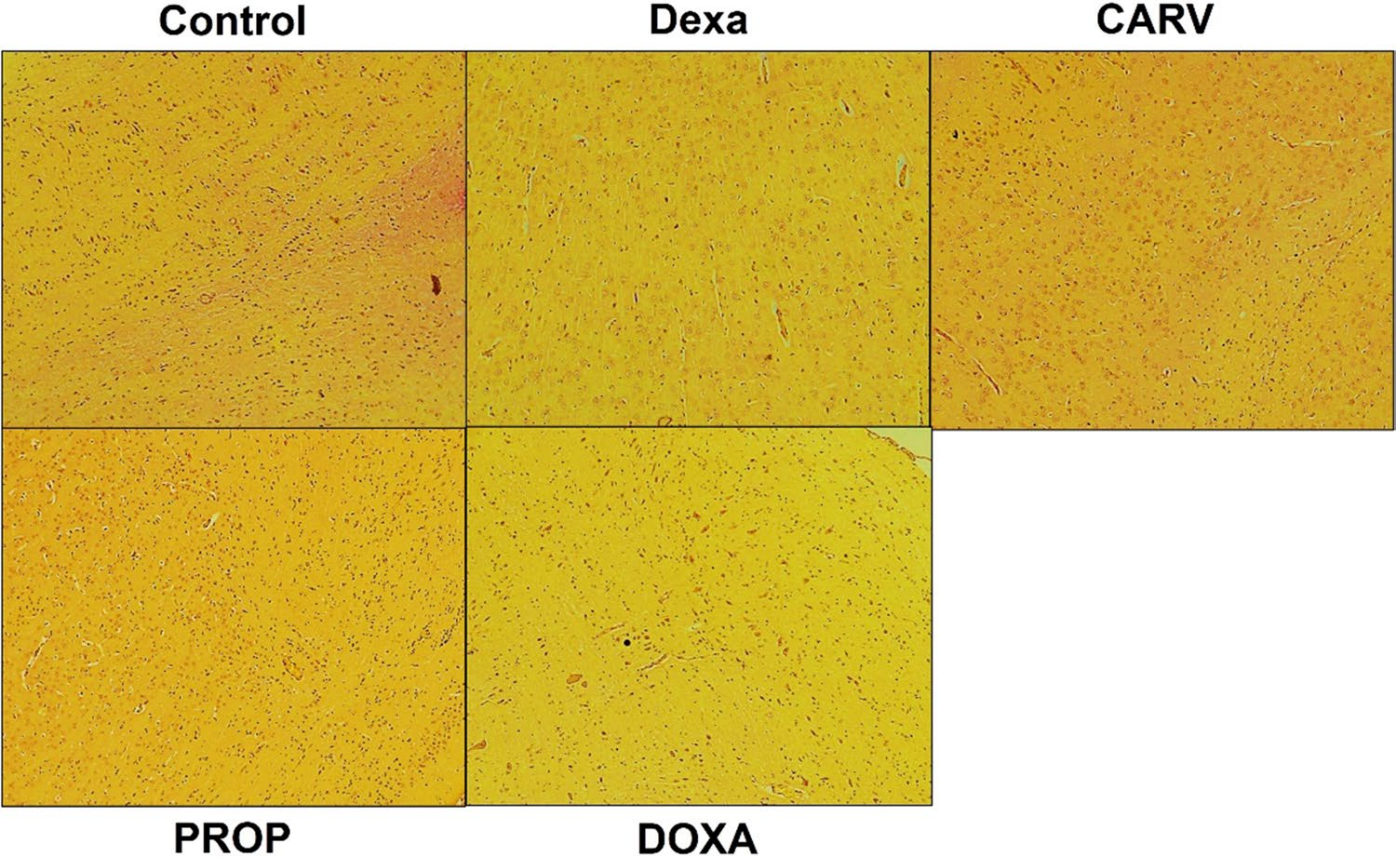
Changes in collagen deposition in the cerebral cortex. Microscopic images of cerebral cortex cross-sections stained with Sirius red (100x). Control: Rats received vehicle for 7 days. DEXA: Rats received dexamethasone (10 mg/kg, S.C.) for 7 days. CARV: Rats received carvedilol (10 mg/kg, I.P.) then dexamethasone 2 hours later for 7 days. PROP: Rats received propranolol (30 mg/kg, I.P.) then dexamethasone 2 hours later for 7 days. DOXA: Rats received doxazosin (5 mg/kg, I.P.) then dexamethasone 2 hours later for 7 days

In Fig. 6, no collagen deposition was detected in all studied groups after staining cerebral cortex sections with Sirius red stain.

#### Carvedilol increased, while doxazosin decreased β-arrestin2 expression in the cerebral cortex of dexamethasone-treated rats

**Fig. 7:**
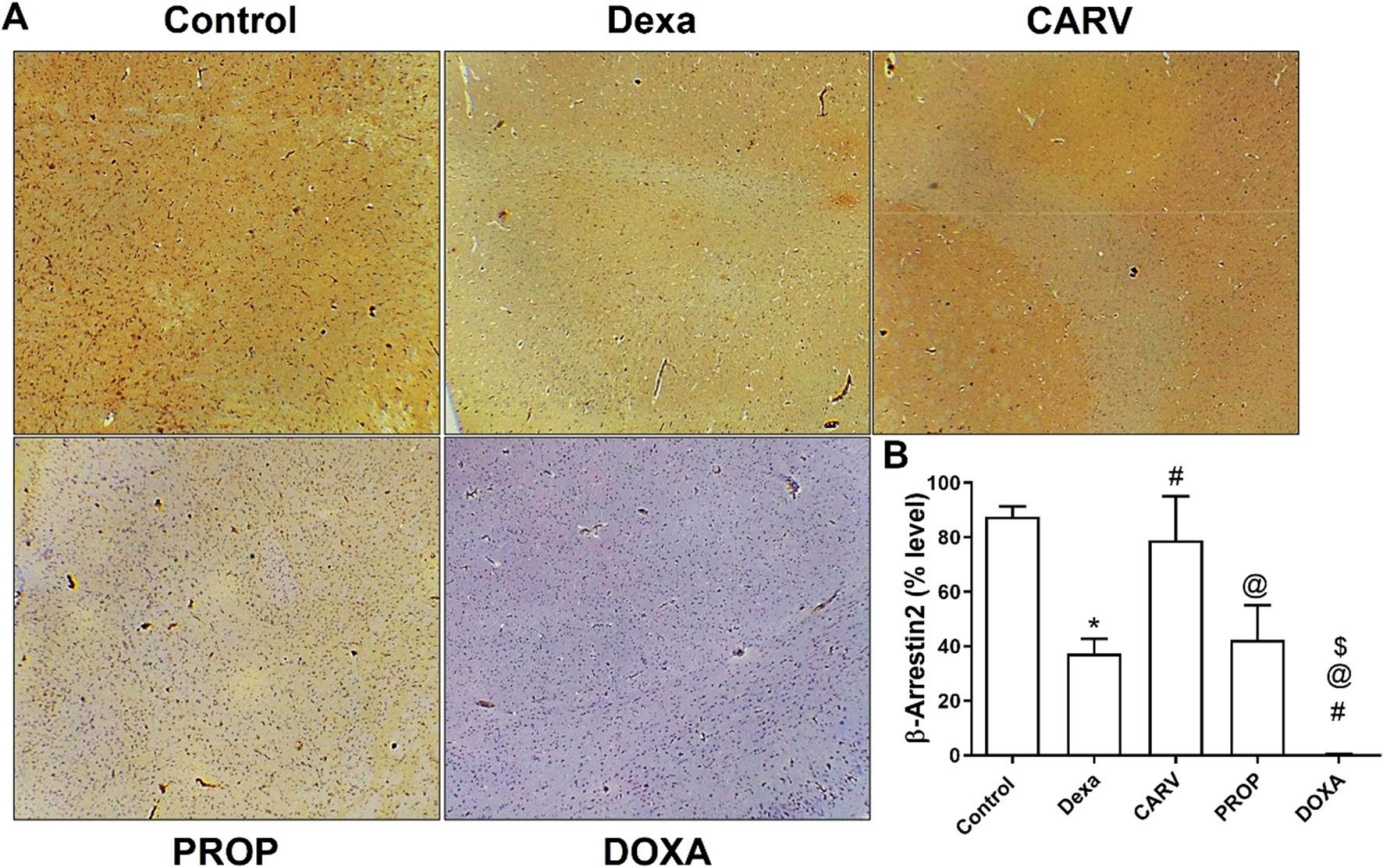
Changes in the cerebral cortex level of β-arrestin2. Microscopic images of cerebral cortex cross-sections immuno-stained using β-arrestin2 monoclonal antibody (100x) (**A**) and graphical presentation of β-arrestin2 level (**B**). Control: Rats received vehicle for 7 days. DEXA: Rats received dexamethasone (10 mg/kg, S.C.) for 7 days. CARV: Rats received carvedilol (10 mg/kg, I.P.) then dexamethasone 2 hours later for 7 days. PROP: Rats received propranolol (30 mg/kg, I.P.) then dexamethasone 2 hours later for 7 days. DOXA: Rats received doxazosin (5 mg/kg, I.P.) then dexamethasone 2 hours later for 7 days. Statistical analysis was performed using one way ANOVA followed by Bonferroni post-test for selected pairs. Values are expressed as mean ± S.E.M. n=3 for all groups. This number represents 3 different cut sections from 3 different rats in each group. * *p* < 0.05 vs Control; # *p* < 0.05 vs Dexa; @ *p* < 0.05 vs CARV; $ *p* < 0.05 vs PROP

As estimated from Fig. 7, dexamethasone treatment significantly decreased β-arrestin2 expression compared to control group (F_4,10_=39.43, *p* < 0.0001). But carvedilol treatment significantly increased β-arrestin2 expression compared to dexamethasone-, propranolol-, and doxazosin- treated rats. Furthermore, doxazosin treatment significantly decreased β-arrestin2 expression compared to dexamethasone- and propranolol-treated rats.

## Discussion

In the present work, subcutaneous injection of dexamethasone (10 mg/kg) for 7 days in rats caused significant increases in the molecular markers of neural injury in the cerebral cortex in addition to marked degeneration of the cortical neurons and disruption of its integrity. On the other hand, treating rats with carvedilol, propranolol and doxazosin ameliorated all molecular changes in the cerebral cortex and slightly reduced the histopathological changes elicited by subcutaneous injection of dexamethasone. However, the three treatments used in the current study induced distinct effects on β-arrestin2 expression, a potential neuroprotective protein. Carvedilol significantly increased, while doxazosin significantly decreased β-arrestin2 expression in the cerebral cortex of dexamethasone-treated rats. On the contrary, propranolol did not induce significant changes in β-arrestin2 expression.

Dexamethasone neurotoxicity has been reported in several previous experimental and clinical studies [30–32]. Intraperitoneal injection of either 7 or 20 mg/kg dexamethasone caused neurotoxicity in the cerebral cortex after 24 hours by increasing the activity of N-methyl D-aspartate (NMDA) receptors and suppression of neurotrophins [30]. In addition, low dose dexamethasone (0.2 mg/kg) acutely induced c-Fos expression in the hippocampus of neonatal rats and increased apoptotic marker expression specifically in the subiculum [31]. In the same context, a previous study showed that a 10-year-old boy who received a 5-week course of glucocorticoids for an acute asthma attack suffered from progressive decline in academic performance and estimated IQ even after long time of discontinuation of glucocorticoid treatment [32].

In accordance with previous findings [30–32], the present study showed that dexamethasone injection for 7 days slightly reduced brain weight and survival rate compared to control group. Also, histopathological examination of the cerebral cortex using H&E and Nissl stains showed marked degeneration in the cortical neurons and disruption of its integrity. In addition, dexamethasone significantly reduced BDNF level and Akt kinase activity in the cerebral cortex. Brain derived neurotrophic factor is a member of neurotrophins that has a vital role in regulating memory in addition to its well-established function in promoting neuronal survival and differentiation [33]. In the same context, Akt is an important survival signal that mediates brain development. Selective downregulation of Akt3, a subtype of Akt proteins, has been found to reduce brain size and weight [34].

On the other hand, dexamethasone significantly increased GFAP and DAG levels in the cerebral cortex compared to control group. GFAP is the main intermediate filament protein in mature astrocytes and a key component of its cytoskeleton during development [35]. Moreover, GFAP expression has been found to increase in the brains of patients suffering from Alzheimer’s disease [36]. In the same context, DAG is an important lipid mediator that can activate several oxidative stress and inflammation signaling. In addition, upregulation of DAG in the cerebral cortex and hippocampus has been found to impair cognitive function [37].

Alpha smooth muscle actin is a subtype of actin proteins that is expressed in vascular smooth muscles and in astrocytes in the central nervous system (CNS) [38]. Expression of α-SMA in astrocytes has been found to be increased in certain types of disorders affecting the CNS such as multiple sclerosis and Alzheimer’s disease [39, 40]. In the latter one α-SMA expression increased in the whole cortex and hippocampus [40]. Like previous findings [39, 40], our results showed significant increases in α-SMA expression in the cerebral cortex of dexamethasone-treated rats compared to control group.

SMAD3 is a component of the transforming growth factor (TGF)-β signaling pathway which is upregulated in response to neural injury as a repairing mechanism. However, SMAD3 can also promote gliosis in addition to being neuroprotective [41]. The present study showed that dexamethasone significantly increased SMAD3 expression in the cerebral cortex compared to control group. In the same context, our results showed that dexamethasone treatment significantly increased the cerebral cortex levels of β-amyloids (1-42) and phospho-Tau protein compared to control group. β-amyloids and phospho-Tau protein are both hallmarks of neurodegenerative disorders such as Alzheimer’s disease. Increased cerebral cortex and hippocampal levels of β-amyloids and phospho-Tau protein were previously recorded in a study investigating the molecular mechanisms of Alzheimer’s disease [42].

Although dexamethasone treatment significantly increased α-SMA level in the cerebral cortex compared to control group, no significant changes were observed in the fibrosis area and collagen deposition. An interpretation of this finding is that secretion of extracellular matrix such as collagen is independent of α-SMA as estimated by a previous study [43]. Furthermore, data available about changes in collagen levels in neurodegenerative disorders is conflicting [44, 45]. A previous study reported a significant increase in collagen deposits in brain tissue of Alzheimer's disease patients [44], in contrast to another study which showed no changes [45].

β-Arrestin2 is an important intracellular protein that mediates desensitization and internalization of GPCRs in addition to initiation of distinct downstream signaling pathways by recruiting and binding intracellular proteins promoting their interaction. Several studies have shown potential neuroprotective effects of β-arrestin2 [11–13]. β-Arrestin2 has been found to protect neurons by mediating endogenous opioid arrest of inflammatory microglia [11]. In addition, amisulpride, an atypical antipsychotic drug and a potent dopamine 2 receptor antagonist, can mediate neuroprotection by activating β-arrestin2 signaling [12]. Furthermore, β-arrestin2 has been found to protect against sevoflurane-induced neuronal apoptosis [13].

On the contrary, other studies showed detrimental effects of β-arrestin2 in the development of neurodegenerative disorders [46, 47]. Overexpression of β-arrestin2 has been shown to be associated with increased β-amyloids generation in Alzheimer’s disease [46]. In addition, β-arrestin2 mRNA levels were elevated in postmortem brain tissue from patients with Alzheimer’s disease compared with age-matched controls [47].

In the same line with the reports that showed β-arrestin2 neuroprotective effects [11–13], dexamethasone-induced neurotoxicity was associated with downregulation of β-arrestin2 in the cerebral cortex compared to control group.

Carvedilol is a 3^rd^ generation β-blocker with weak α1-blocking effects. In addition, carvedilol has unique β-arrestin biased agonistic effects, anti-inflammatory, and antioxidant effects [14]. Carvedilol is a moderately lipophilic drug that can cross the blood brain barrier promoting central effects [48]. Previously, carvedilol has been reported to mediate neuroprotective effects in an experimental model of brain ischemia by reducing production of reactive oxygen species (ROS) [17]. In addition, carvedilol showed antioxidant and neuroprotective effects against rat model of diabetic neuropathy [18]. In the same context, carvedilol showed antidepressant effects associated with increased BDNF levels in the cortex and hippocampus of mice exposed to chronic unpredictable stress [49]. Furthermore, carvedilol has shown promising therapeutic effects against Alzheimer’s disease [50]. Chronic oral administration of carvedilol reduced β-amyloids production and cognitive deterioration in two different models of Alzheimer’s disease [51].

In harmony with previous findings [17, 18, 49, 50], the present study showed that carvedilol increased survival rate, slightly reduced the histopathological changes, and significantly increased brain weight, BDNF, Akt kinase activity, and β-arrestin2 levels in the cerebral cortex compared to the dexamethasone group reflecting neuroprotective effects. Moreover, carvedilol significantly decreased GFAP, DAG, α-SMA, SMAD3, β-amyloids (1-42) and phospho-Tau protein levels compared to the dexamethasone group reflecting alleviation of dexamethasone-induced neurotoxicity.

Propranolol is a highly lipophilic non-selective β-blocker that can cross the blood brain barrier and can show weak β-arrestin agonistic effects [51, 52]. Previously, propranolol has shown neuroprotective effect against retinal degeneration in a mouse model of light injury [19]. Moreover, propranolol reduced all behavioral and molecular markers of neural injury in a genetic mice model of Alzheimer's disease [53]. Propranolol treatment for 6 weeks at a dose of 5mg/kg reduced cognitive impairments and hippocampal β-amyloids and phospho-Tau protein levels and increased BDNF and Akt activity in diseased mice [53].

In harmony with previous findings [19, 53], our results showed that propranolol significantly increased BDNF level and Akt kinase activity in the cerebral cortex compared to the dexamethasone group reflecting neuroprotective effects. Also, propranolol slightly reduced the histopathological changes and significantly decreased GFAP, DAG, α-SMA, SMAD3, β-amyloids (1-42) and phospho-Tau protein levels compared to the dexamethasone group reflecting alleviation of dexamethasone-induced neurotoxicity. Notably, propranolol did not change β-arrestin2 level compared to the dexamethasone group. The latter finding shows that propranolol neuroprotective effects may be independent of β-arrestin2 signaling.

Doxazosin is a selective α1-AR blocker that can cross the blood brain barrier mediating central effects [54]. Previously, doxazosin has been reported to protect against neuroblastoma by inhibiting Akt activity leading to apoptosis of undifferentiated neuroblastoma cells [20]. On the contrary, doxazosin increased Akt activity in differentiated nerve cells making it a tool in the management of neurodegenerative disorders [20]. In this context, doxazosin showed neuroprotective effects in an in vitro model of Alzheimer's disease characterized by increased Akt activity, reduced phosphorylation of Tau proteins, and reduced neurotoxic effects of β-amyloids on hippocampal slices [20].

The present study showed that doxazosin significantly increased BDNF level and Akt kinase activity in the cerebral cortex compared to the dexamethasone group reflecting neuroprotective effects. In addition, doxazosin slightly reduced the histopathological changes and significantly decreased GFAP, DAG, α-SMA, SMAD3, β-amyloids (1-42) and phospho-Tau protein levels compared to the dexamethasone group reflecting alleviation of dexamethasone-induced neurotoxicity. Noteworthy, doxazosin significantly decreased β-arrestin2 level in the cerebral cortex compared to the dexamethasone group. Although carvedilol neuroprotective effects were associated with upregulation of β-arrestin2 level in the cerebral cortex, doxazosin showed the reverse. This may reflect multifunction and distinct effects of β-arrestin2 in the nerve cell pathophysiology which may need further investigations to understand the underlying mechanisms.

Considering the changes in the molecular markers of neural injury, both carvedilol and propranolol showed nearly the same neuroprotective effects against dexamethasone-induced neurotoxicity. However, doxazosin was the most potent neuroprotective agent in this model. Notably, the doses of carvedilol and propranolol used in the current study are pharmacologically equivalent based on their anti-hypertensive effects [25].

On the other hand, the dose of doxazosin used in the current study may be much higher than those of carvedilol and propranolol doses regarding the anti-hypertensive effect [27]. However, it is not clear if this variation in neuroprotection is related to the difference in dose levels. This point needs further investigations. Also, it is not clear whether the neuroprotective effects of the used drugs can be achieved by other types of β- and α1-AR blockers or not.

In conclusion, we showed for the first time, to our knowledge, that subcutaneous injection of dexamethasone (10 mg/kg) for 7 days in rats induces neurotoxicity in the cerebral cortex that is similar in its molecular and histopathological patterns to that of neurodegenerative disorders such as Alzheimer’s disease. Furthermore, blocking β- and/or α1ARs by using either carvedilol, propranolol, or doxazosin alleviate dexamethasone-induced neurotoxicity. However, changes in β-arrestin2 level are distinct among different treatments and may reflect multifaceted roles of β-arrestin2 in nerve cell pathophysiology.

## Data Availability

All the relevant data are reported within the paper. For additional details, data are available on request to the authors.
